# Special Issue: “The Latest Clinical Advances in Thrombocytopenia”

**DOI:** 10.3390/jcm10163463

**Published:** 2021-08-05

**Authors:** Hugo ten Cate, Bernhard Lämmle

**Affiliations:** 1 Thrombosis Expertise Center, Cardiovascular Research Institute Maastricht (CARIM), Maastricht University Medical Center, NL-P.O. Box 616, 6200 MD Maastricht, The Netherlands; 2 Departments of Internal medicine and Biochemistry, Maastricht University Medical Center, NL-P.O. Box 616, 6200 MD Maastricht, The Netherlands; 3 Center for Thrombosis and Hemostasis, University Medical Center Mainz, Johannes Gutenberg University, 55131 Mainz, Germany; bernhard.laemmle@uni-mainz.de; 4 Central Hematology Laboratory, Department of Hematology, Inselspital, Bern University Hospital, University of Bern, CH 3010 Bern, Switzerland; 5 Haemostasis Research Unit, University College London, London WC1E 6BT, UK; 6 Schützenweg 3, CH 3065 Bolligen, Switzerland

Platelets are critical elements in the blood stream, supporting hemostasis as well as performing even more complex tasks within networks of biological (immunity) and pathophysiological processes, such as cancer and ischemia/reperfusion injury. Changes in the number (and function) of platelets may have a substantial impact on any of these processes. The “simple” finding of a reduced platelet count (thrombocytopenia) has a history (origin) and a consequence (e.g., bleeding). The origin of thrombocytopenia can be unclear (idiopathic), can depend on associated illness (e.g., disseminated intravascular coagulation, heparin-induced thrombocytopenia, thrombotic thrombocytopenic purpura) with a different etiology depending on the illness (related to production, clustering, immune depletion, intravascular consumption, etc.), and can also be acquired (e.g., during extracorporeal circuits, such as “ECMO”) or constitutional. In the current COVID-19 pandemic, thrombocytopenia is also a feature, although it is less severe than in other diseases, and the overall patient phenotype seems thrombotic rather than hemorrhagic. Thus, there are many possible causes for thrombocytopenia, and these are, quite often, poorly characterized. Since the clinical question is always whether or not the platelet deficit has consequences for the patient, it is important, and also timely (e.g., COVID-19), to discuss this topic in a Special Issue.

We, the editors, are extremely pleased that a large number of outstanding clinician-scientists were willing to contribute to this Special Issue! In times of substantial stress due to the COVID-19-related burden on society and work, the request to contribute a state-of-the-art paper for any journal is a major demand for any author, junior or experienced. To comply with strict timelines is a further challenge.

We consider ourselves very lucky that the authors we approached were, without exception, willing to do their best and to make it before the deadline. We are proud of the high quality of the papers we received. Now that this series is complete, at least according to our expectations, we hope that the readership will enjoy it. We observe that a number of these papers are already viewed at a high rate and even cited, supporting the value of this article series. For this issue, we acquired one original research article [1] and 13 reviews [2,3,4,5,6,7,8,9,10,11,12,13,14] that are shortly introduced below.

Falter, Böschen, Schepers, Beutel, Lackner, Scharrer, and Lämmle report their final analysis on a questionnaire study from 2015 and 2016 into neurocognitive and mental sequelae on 104 adult patients having survived at least one acute episode of autoimmune thrombotic thrombocytopenic purpura (iTTP) at the University Medical Center in Mainz or having consulted with this center [1]. Data on depressive symptoms and cognitive deficits in this cohort obtained in 2013 and 2014 had already been published before. Based on self-reporting using a series of validated questionnaires, iTTP survivors had a high prevalence of depression and anxiety, a more negative attitude to life, and low resilience as compared to controls. Quality of life and cognitive performance in patients were significantly reduced (see also the review article on thrombotic thrombocytopenic purpura by Sukumar et al. [3], introduced below).

Bury, Falcinelli, and Gresele comprehensively review the current knowledge of the inherited thrombocytopenias (ITs) [2]. As of today, more than 40 different disorders caused by defects of various genes involved in megakaryopoiesis and platelet production are known, and the field is rapidly evolving, not least by the diagnostic advances supported by modern next-generation sequencing techniques. Some ITs show isolated thrombocytopenia, others are associated with various clinical features (so-called syndromic ITs) and, importantly, some forms are associated with a tendency to develop a hematologic neoplasm during ensuing years. Bleeding diathesis is highly variable and generally more severe in ITs with associated functional platelet defects. Even though the general internist and hematologist may not have full knowledge on all individual ITs, it is mandatory to think of the possibility of a hereditary thrombocytopenia by obtaining a complete personal and family history and (in unclear cases) to proceed to genetic screening early in the diagnostic work-up in order to avoid misdiagnosis of immune thrombocytopenia (ITP) and unnecessary, potentially harmful immunosuppressive treatment. The ethical concerns of uncovering a potential premalignant condition are stressed by the authors.

Sukumar, Lämmle, and Cataland present an update on thrombotic thrombocytopenic purpura (TTP), a rare but potentially fatal disease caused by a severe autoantibody-mediated (iTTP) or congenital deficiency (cTTP) of the metalloprotease ADAMTS13 [3]. ADAMTS13 deficiency is associated with a defective proteolytic processing of the Von Willebrand factor (VWF), leading to the presence of unusually large, extremely adhesive VWF multimers in plasma and spontaneous widespread platelet clumping in the microcirculation. Severe thrombocytopenia and microangiopathic hemolytic anemia caused by erythrocyte fragmentation in the partially occluded microcirculation as well as ischemic organ dysfunction can rapidly lead to death if untreated. Besides, the established therapy with large-volume plasma exchange, fresh frozen plasma replacement and corticosteroids, new adjunctive therapeutic modalities with caplacizumab, inhibiting VWF–platelet interaction, and with the anti-CD20 antibody rituximab, inhibiting autoantibody production in iTTP, are described. Recombinant ADAMTS13 as a replacement for patients with cTTP is under investigation. Regular follow-up of iTTP and cTTP patients is imperative to minimize long-term sequelae (see also the article by Falter et al. [1] mentioned above). 

Lardinois, Favresse, Chatelain, Lippi, and Mullier—starting from an observed case—review the not uncommon condition known as pseudothrombocytopenia (PTCP) [4]. It is of utmost relevance to recognize this laboratory phenomenon of an apparently decreased platelet count due to in vitro platelet clumping in order to avoid unnecessary and potentially harmful diagnostic and therapeutic measures. Best known is EDTA-induced PTCP, but multi-anticoagulant PTCP does exist, and they are best recognized by visual inspection of a peripheral blood smear. Of note, no specific disease is associated with or heralded by PTCP, even in patients followed for many years. Lardinois et al. provide a detailed discussion on the pathomechanism, the laboratory diagnostic approach including the use, if available, of various platelet counting techniques, different in vitro anticoagulants, and other analytical considerations. This overview is especially useful for the hematologic laboratory specialists confronted with this sometimes-challenging condition.

Squiccimarro, Jiritano, Serraino, ten Cate, Paparella, and Lorusso contribute a review on the quantitative and qualitative platelet derangements in cardiac surgery and extracorporeal life support [5]. Thrombocytopenia and simultaneous functional abnormalities of platelets are common in cardiac surgery using cardiopulmonary bypass (CPB) and extracorporeal membrane oxygenation (ECMO). The pathophysiologic mechanisms and complex interactions of platelets, other blood cells, activation of coagulation and complement systems, and release of cytokines and chemokines associated with heart surgery while blood is flowing over artificial surfaces are outlined. The need of heparinization may pose a high risk of heparin-induced thrombocytopenia for cardiac surgery patients (see also the review by Marchetti et al. [6] mentioned below). 

Marchetti, Zermatten, Bertaggia Calderara, Aliotta, and Alberio review the still common and highly dangerous prothrombotic heparin-induced thrombocytopenia (HIT) [6]. They provide an update on the pathophysiology of this immunologic syndrome, which may be a “side effect” of a host immune mechanism intended to fight against Gram-negative bacterial infections. HIT pathophysiology involves monocytes, endothelial cells, and neutrophils, as well as platelets. The necessity of a Bayesian combined clinical and laboratory approach to diagnosis is stressed. Assessing clinical pretest probability and a combination of fast (semiquantitative) immunoassays may massively shorten diagnostic work-up and obviate the need for emergency testing of functional HIT antibodies. Finally, besides the current established treatment, emerging therapeutic concepts are discussed, including the difficult situation of cardiovascular surgery in patients with HIT or a history of HIT. The Lausanne HIT team exemplarily shows that each large medical center should have an established algorithm for the awareness and management of this condition.

Singh, Uzun, and Bakchoul provide an interesting review on the pathophysiology, diagnosis, and management of immune thrombocytopenia (ITP) [7]. They stress the multifactorial pathogenesis of this common condition, including the increased platelet sequestration and destruction as well as impaired platelet production. Autoantibodies against platelet glycoproteins lead to opsonization of platelets which are removed via Fc receptors on macrophages predominantly in the spleen. Autoantibodies may also promote de-sialylation of platelet glycoproteins, rendering these platelets susceptible to removal via the Ashwell-Morell receptors in the liver and, importantly, platelet production is compromised by autoantibodies. The authors suggest measuring anti-platelet glycoprotein autoantibodies with appropriate tests during initial diagnostic work-up even though current guidelines provide no firm recommendation. Treatment modalities are extensively discussed and some new drugs currently under investigation are presented as well.

Raadsen, Du Toit, Langerak, Van Bussel, Van Gorp, and Goeijenbier present a broad overview of the literature from the past 10 years on the relation/interaction of “platelets” with “viral infections” [8]. They complemented their extensive literature analysis by a second search performed in December 2020, focusing on the publications of the preceding year on SARS-CoV-2 and platelets. The role of platelets far exceeds the well-known hemostatic function, and they seem to have direct and indirect antiviral activity and are involved in the accompanying immunologic reactions. A large list of specific viral infections is discussed with highly varying degrees of thrombocytopenia. The most severe viral hemorrhagic fevers, e.g., Ebola, Marburg, and Lassavirus infections, are under-investigated because of the scarcity of laboratories with biosafety level 4 worldwide. Based on the current COVID-19 pandemic, the authors stress that any immunomodulating therapy potentially affecting platelets must be carefully evaluated because the immunothrombosis mechanisms may serve an important antiviral role which should not be compromised. 

Aliotta, Bertaggia Calderara, Zermatten, Marchetti, and Alberio offer an in-depth review on thrombocytopathies [9]. They first outline the platelet activation endpoints, namely adhesion, secretion, and aggregation, discuss their classic defects such as Bernard-Soulier syndrome, platelet-type von Willebrand disease, alpha- and delta-storage pool deficiencies, and Glanzmann thrombasthenia respectively, and then highlight the major role of platelet procoagulant activity. The latter is primarily provided by the exposure of phosphatidylserine on the outer membrane of the phospholipid bilayer and the release of platelet microparticles. The technically demanding and difficult to standardize methodologies of assaying platelet procoagulant functions are explained. It is demonstrated that a low or high potential to generate procoagulant platelets in vitro may be associated with bleeding or thrombotic tendencies, respectively. A short summary of the thrombocytopathy associated with COVID-19 is added (for a discussion of platelet function testing, specifically in thrombocytopenic patients, see also the review by Jurk and Shiravand [11] introduced below).

Bonadies, Rovo, Porret, and Bacher expertly discuss thrombocytopenia in the context of hematologic diseases, i.e., myelodysplastic syndromes (MDS) and bone marrow failure syndromes (BMF) [10]. They describe the variable clinical presentations of patients with MDS and BMF, the latter consisting of acquired aplastic anemia and inherited forms of BMF, such as Fanconi anemia and several telomeropathies. The authors provide a detailed overview on the often-stepwise diagnostic approach, including cytologic investigation of peripheral blood and bone marrow aspirate, histopathology of bone marrow, flow cytometric immunophenotyping, and cytogenetic and next-generation sequencing analyses, obviously requiring specialized multidisciplinary teams. The authors remind us that an isolated thrombocytopenia, especially in the elderly, may herald either an evolving MDS or an aplastic anemia, and they stress the importance, especially in children, to consider an inherited BMF syndrome (see also the review by Bury et al. [2] in this issue for inherited thrombocytopenias predisposing to MDS and/or leukemia).

Jurk and Shiravand share their expertise on platelet phenotyping and function testing in thrombocytopenic patients [11]. Patients with inherited or acquired thrombocytopenia may additionally display functional platelet defects, contributing to the bleeding risk. The indications for such platelet function testing in thrombocytopenia are presented. The evaluation should start with a standardized assessment of the bleeding history and of the basic hematologic parameters, including inspection of a peripheral blood smear. The frequently used point-of-care tests (Multiplate^®^ analyzer, Platelet function analyzer PFA-100^®^ or PFA-200^®^, Impact-R™, thromboelastography (TEG^®^), and rotational thromboelastometry (ROTEM^®^)) assess the global primary (and the latter two also the secondary) hemostasis and are not suitable to diagnose platelet dysfunction in thrombocytopenia. Light transmission aggregometry, lumi-aggregometry, flow cytometric platelet phenotyping and functional testing in vitro, and assay of platelet-dependent thrombin generation by calibrated automated thrombinography (CAT) allow the detailed characterization of platelet function defects. All these latter assays require expertise and are reserved for specialized laboratories. The same applies to microfluidic flow chamber assays of thrombus formation used for research purposes (see also the review by Aliotta et al. [9] introduced above).

Leader, Hofstetter, and Spectre provide an overview on the difficult topic of managing thrombocytopenic cancer patients by non-transfusion-based means [12]. Thrombocytopenia in patients with malignant neoplasms may be chemotherapy-induced or related to the malignant disease, e.g., in myelodysplastic syndromes (MDS) or acute leukemia (AML). First, the prophylactic and therapeutic roles of antifibrinolytic treatment with tranexamic acid and epsilon-amino caproic acid in various tumors are presented. Second, the efficacy and safety of thrombopoietin receptor agonists are outlined and results of performed and ongoing trials in MDS/AML and in chemotherapy-induced thrombocytopenia in solid tumors are summarized. Finally, the major dilemma of managing antithrombotic therapy with anticoagulants and/or antiplatelet agents in thrombocytopenic cancer patients who are often at high risk of both thromboembolic and bleeding complications is discussed. The authors also refer to the need for platelet transfusions in specific situations (see also the review by Capraru et al. [14], mentioned below). 

Scharf provides an overview on thrombocytopenia, platelet dysfunction, and overall hemostatic changes in acute and chronic liver disease [13]. Thrombocytopenia is common and has a multifactorial pathophysiology in hepatopathies, involving splenomegaly with splenic sequestation of platelets, reduced hepatic thrombopoietin production, and increased platelet destruction. Plasmatic coagulation is impaired as well but is “rebalanced” by a concordant reduction of pro- and anti-hemostatic factors. The resulting low-level hemostatic balance predisposes not only to bleeding but also to thrombotic complications. Management of thrombocytopenia in liver disease may profit from newly available thrombopoietin receptor agonists, which allow to avoid platelet transfusions in many instances. Limitations, risks, benefits, and general concepts for optimal hemotherapy of patients with liver disease are outlined.

Capraru, Jalowiec, Medri, Daskalakis, Zeerleder, and Taleghani review the current state of platelet transfusion and provide an outlook on ongoing and future developments from the perspective of transfusion medicine [14]. The authors explain the differences between apheresis platelet concentrates from single donors and pooled platelet concentrates from buffy coat or platelet-rich plasma. Next, storage media (platelet additive solutions) intended to increase the shelf life of platelet concentrates and reduce allergic reactions to contaminating plasma are discussed. Then, pathogen inactivation technologies aiming at decreasing the transmission of bacterial and viral infections by platelet transfusion are outlined. Cold storage (at 4 °C), cryopreservation (at −80 °C), and even lyophilization of thrombocyte preparations for transfusion are being studied. Finally, the authors discuss a series of alternatives to platelet transfusion that are under exploration, such as “thromboerythrocytes”, “plateletsomes”, in vitro production of platelets from pluripotent stem cells, and others, highlighting a very active research area in this field of transfusion medicine.

We hope that this compilation of articles and reviews on various pathophysiologic, diagnostic, and therapeutic aspects of thrombocytopenia is useful for clinicians and researchers from different specialities.
